# PReS-FINAL-1015: A systematic literature review on diagnosis and treatment of pediatric rheumatic diseases: a shared initiative

**DOI:** 10.1186/1546-0096-11-S2-P12

**Published:** 2013-12-05

**Authors:** V Boom, N de Graeff, F Belutti Enders, N Groot, N ter Haar, F Sperotto, S Vastert, N Wulffraat

**Affiliations:** 1 Department of Pediatrics of the Wilhemina Children's Hospital, University Medical Centre Utrecht (UMCU), Utrecht, The Netherlands; 2 Department of Pediatrics of the Wilhemina Children's Hospital, University Medical Centre University Medical Centre Utrecht (UMCU), Utrecht, The Netherlands; 3 Alder Hey Children's NHS Foundation Trust Hospital, Liverpool, UK; 4 University of Padua, Department of Pediatrics, Padova, Italy

## Introduction

Pediatric Rheumatic Diseases (PRD) represent a group of rare diseases that can lead to significant morbidity. As is the problem with many rare diseases, evidence-based guidelines are lacking and treatment is mostly based on physician experience. Consequently, treatment regiments differ throughout Europe. This year, a new project called SHARE (Single Hub and Access point for pediatric Rheumatology in Europe) was launched to describe what is needed for optimal diagnosis and treatment for children and young adults with rheumatic disease. This project tackles problems across different fields, ranging from access to resources to ethical consideration to quality and uniformity of health care.

## Objectives

As a part of SHARE, a work package has been defined to identify best practices and establish minimal standards of care for the treatment of patients suffering from PRD, in order to improve and standardize care across Europe.

## Methods

A systematic review was conducted on specific questions regarding diagnosis, treatment and complications of PRD, i.e. Juvenile Idopathic Artritis, childhood-onset Systemic Lupus Erythematosus, Anti Phospholipid Syndrome, vasculitis, scleroderma, juvenile dermatomyositis and Periodic Fever Syndromes. Articles from 1970 onwards were included. Related articles on MEDLINE, EMBASE and Cochrane were selected using systematically built and validated search strings, yielding more than 30.000 hits. Reviews, case-reports and case-series smaller than three cases were excluded. After screening, this number of papers will be reduced to several thousands and a review process will be executed according to EULAR guidelines by groups of experts from PReS workgroups.

## Results

The results from the systematic reviews will form the basis of guidelines on minimal standard of care. Consensus meetings will finalize these guidelines by filling in the shortcomings of existing evidence with expert opinion, using the Delphi method. The final result of this work package will be the formulation of minimum standards of care per individual PRD.

## Conclusion

It is essential to formulate well-founded standards of care for these rare pediatric diseases; doing so will most importantly benefit patients themselves, but also increase uniformity of care within the European Union. All in all, SHARE will thus facilitate improved and more uniform care within Europe.

## Disclosure of interest

None declared.

